# Dr. Adams's Remarks on Mr. Moore's History of Vaccination

**Published:** 1818-04

**Authors:** 


THE LONDON

### Medical and Physical Journal.

4 OF VOL. XXXIX.] APRIL, 1818. [NO. 230.

"For many fortunate discoveries in medicine, and for the detection of nume-"rous errors, the world is indebted to the rapid circulation of Monthly "Journals; and there never existed any work to which the Faculty in "EUROPE and AMERICA were under deeper obligations than to the "Medical and Physical Journal of London, now forming a long, but an "invaluable, series."—RUSH.

*For the London Medical and Physical Journal.*

### Dr. ADAMS's *Remarks on Mr. Moore's History of Vaccination.*

THE last Retrospect of the Journal contains an expression of regret, that Mr. Moore had revived animosities which might otherwise have ceased. Of these, I shall take no other notice than to request an explanation of some passages in the few pages of the second chapter, which seem to be directed personally to me.

"CHAP. II.—*The Promulgation of the Vaccine in England, and the invidious Conduct of some early Proselytes.*

"From the time in which Jenner, when a youthful medical student, first heard the country rumours respecting the cow-pox, until the period in which he completed the discovery of vaccination, he never concealed his progressive knowledge; but openly divulged it to his friends, and even to medical societies. The original report he had mentioned to Mr. Hunter, in the year 1770, when he resided with him; and, in two years after, when on a visit to London,* he showed him a drawing of a finger affected with the vaccine; and proposed, at that time, vaccination as a substitute for variolous inoculation. In consequence of this disclosure,† a pupil of Mr. Hunter's noticed, in a publication, that the cow-pox was a preventive of small-pox: this was copied by others, and was also mentioned in medical lectures in London.

"Notwithstanding all this publicity, Jenner was in no point

"* Evidence at large, as laid before the Committee of the House of Commons, &c. London, 1803. Vide pages 1, 11, 135."

"† Queries concerning Inoculation, by Dr. Beddoes, 1795.—Observations on Morbid Poisons, by J. Adams, 1795, p. 156.—History of Inoculation, by Woodville, A.D. 1796; Introduction, note, p. 3."

Dates in historical accounts are important. The inaccuracy of the above will presently be shown.—A.

anticipated, and his opinions were commonly regarded as the reveries of a rural enthusiast. It may, therefore, be presumed, that it required his peculiar cast of character to perfect the discovery: and, it is certain, that if, by an alteration in the management of the dairies, the vaccine had vanished, the discovery would have become impossible; and the small-pox might have continued its ravages to the end of the world.

"In June 1798, Dr. Jenner being satisfied with the result of his experiments, resolved to lay them before the public. And, as he was a fellow of the Royal Society, and accustomed to divulge his observations in science through that channel, he transmitted his manuscript to a correspondent who was in the confidence of Sir Joseph Banks, the president; and requested that it should be laid before him, not doubting that it would soon be printed in the Philosophical Transactions. Jenner had already contributed several articles to that celebrated collection: in one of these, he had fully disclosed the natural history of the cuckow, which marked him out for a man of originality; and, as none of his former papers on subjects of mere philosophical curiosity had been rejected, he naturally expected, that an essay promulgating a discovery of vast utility, would be favourably received. But the perusal of his experiments produced no conviction; and he received, in reply, a friendly admonition that, as he had gained some reputation by his former papers to the Royal Society,* it was advisable not to present this, lest it should injure his established credit. This advice, though given with the best design, was neglected with the happiest consequences; for, although disappointed in his favourite mode of ushering his discovery into the world, he was confident that his work required no patronage: and, therefore, after the addition of a few experiments made in this interval, he sent to the press his *Inquiry into the Causes and Effects of the Variolæ Vaccinæ, a Disease discovered in some of the Western Counties of England, particularly Gloucestershire, and known by the Name of the Cow-pox.*"

It appears to me very unlikely, that Dr. Jenner should defer the public communication of so valuable a blessing from the year 1770 to 1798, a period of twenty-eight years. I have not the evidence at large to refer to, but this I can assert, that, in the year 1782, Mr. Hunter made no mention of cow-pox in his lectures.

The connexion of the text with the notes in Mr. Moore's work, points out *me as the "pupil of Mr. Hunter."* What then can be the meaning of such an anachronism as we meet with in the second note? Dr. Beddoes' queries† are placed

"* Letter from Dr. Jenner in possession of the author."

† I presume Mr. Moore can only refer to "Contributions, chiefly from the West of England," 8vo. 1799; which contains a set of queries to Mr. Jones, and the answers.

first in order of three publications, though it is certain, that they should stand last, and that they were not published till five years after the first edition of "Adams on Morbid Poisons,"* two years after Woodville's "History of Inoculation, and one year after Jenner's "Inquiry." The order, therefore, should be, J. Adams on Morbid Poisons, 1795—Woodville on Inoculation, 1796—Jenner's "Inquiry," [his first publication on Cow-pox,] 1798—and Beddoes' "Queries," 1799. But, though my name stands first in the order of publication on the cow-pox, I again repeat what I have taken half-a-dozen opportunities of acknowledging, that I knew nothing of the fact, excepting from Dr. Jenner, through the intervention of Mr. Cline. Not only on Dr. Jenner's account do I wish this to be well understood, but on my own: for, engaged as I was in researches concerning Morbid Poisons, it would have been inexcusable in me to have left the enquiry into this valuable prophylactic unfinished, had not Mr. Cline informed me that Dr. Jenner was at that time engaged in experiments on the subject. For the same reason, it is impossible to conceive, that one who has so much contributed to the benefit of mankind would have suffered his discovery to have remained useless for the length of time stated by Mr. Moore.

Now, as to the Royal Society.—I have taken much pains to enquire of Sir Joseph Banks, the president, and of Mr. Lee, the clerk, whether any such papers as mentioned by Mr. Moore are contained among the archives of that institution.—Sir Joseph assures me he does not recollect to have heard of them; and Mr. Lee, that there is nothing of the kind among their archives. The Right Honourable President, therefore, concludes, that they were submitted to an individual member,—probably Mr. Hunter, and at an earlier date than Mr. Moore mentions.

Hence Jenner was, as Mr. Moore observes, no way anti-

---

* The following is extracted from the first edition of Morbid Poisons, published in the year 1795:—"The cow-pox is a disease well known to the dairy-farmers in Gloucestershire. The only appearance on the animal is a phagædenic ulcer on the teat, with apparent inflammation. When communicated to the human subject, it produces, besides ulceration on the hand, a considerable tumour of the arm, with symptomatic fever, both which gradually subside.—What is still more extraordinary, *as far as facts have been hitherto ascertained*, the person who has been infected is rendered insensible to the variolous poison."

cipated, excepting in publication by myself and Woodville. But those publications produced, as it was wished by their authors, no effect on the public mind. This honour was reserved by us both for the Discoverer, who was treated by neither of us as "a rural enthusiast," but considered by both of us as one from whose further investigations the greatest benefit to mankind might be expected.

If Mr. Hunter knew of this discovery, and had received the account from a character so well known to, and so much respected by him, as Dr. Jenner, I think he would scarcely have omitted to produce such a fact in corroboration of his theory concerning the origin of some morbid poisons. In his Treatise on the Venereal Disease, he evidently refers to the probability of that poison originating in some other animal; and, had he known of the cow-pox in 1788, when his second edition was published, there cannot, I think, be a doubt, that he would gladly have corroborated his theory by so interesting and well-ascertained a fact: for, whatever doubts he might entertain of the prophylactic powers of cow-pox, he could entertain none of its local action as a morbid poison in the human subject originating from a brute. It was, probably, not long before his death, that he was made acquainted with the fact, and that the advice he gave was, that the experiment should have its fair trial on the human race before it was published. This is the more likely, as such a proposal would be exactly consonant to his own proceedings: for, in all that he published, excepting his Treatise on the Digestion of the Stomach, (which, he pleads, was prematurely forced from him,) we find nothing unfinished, which could be proved by experiment. I may even be allowed to suggest further, that, had Mr. Hunter lived to see the promulgation of this invaluable discovery, he would have counselled his disciple to reserve his opinion concerning the horse's heel, until he had reduced it to experimental certainty.

May I be allowed to offer a similar defence of my departed friend, Woodville. Nothing but a proper respect to Jenner's claims, could excuse his backwardness in affording the public all the benefits of vaccination as soon as he was made acquainted with it by my publication. The number of experiments he made in the Small-pox Hospital on whatever was suggested, shows his anxiety to improve a practice to which he had devoted himself; but he considered, like me, that the discovery was Dr. Jenner's, and that it would be an intrusion on his property to interfere with it farther than the discoverer had promulgated. That such were his feelings, is evident by Mr. Moore's history; for, no sooner had Dr. Jenner published, than Woodville, considering the subject no longer *privati juris*, and finding he could not procure the fluid from Gloucestershire, immediately searched the neighbourhood of London till he discovered the Cow-pox.* Of the controversy, which is so minutely detailed in the rest of the chapter, it does not become me to take any notice, especially as during the whole time I was at a distance from the scene of action. But Mr. Moore has thought proper to follow me even there.—"In Madeira, (says he, p. 36,) a physician described the vaccine as a *pustular* disease." It would, at least, have been fair to have mentioned the work, if not the passage, in which this description was given. Of the author, there can be no doubt, as no physician in Madeira, excepting Dr. Adams, wrote at that time on the subject.† Now, in all my papers, particular care is taken to show, that the true vaccine poison, when following its legitimate course, never excites the secretion of *pus*, and, consequently, can never be called *pustular*. Even the reason is assigned in the invariable and only distinction between the variolous pustule and the vaccine vesicle: the former characterized by a slough, the separation of which is effected, as in all other cases, by suppuration, and thus becomes the cause of the secretion of pus; the cow-pox, on the contrary, never inducing slough, has no suppuration, consequently NO PUSTULES.

---

* The following are Mr. Moore's words:—"It [the cow-pox] had become extinct, and the cows in Gloucestershire were at that time free from the malady. Woodville then searched the neighbourhood of London, and *accidentally* found the disease in a dairy in Gray's-inn-lane, from which source he commenced a series of experiments at the Small-pox Hospital."—*Moore's History*, p. 26.

*Accident* or *chance* are words not admitted in philosophical reasoning. Roscoe, in an ingenious oration to his townsmen, on the opening of the Liverpool Institution, makes the following very just remark on this subject:—"Hume, says he, doubts whether the rise and progress of the arts are not rather to be attributed to *chance*; as if chance meant any thing more than causes, which it is difficult, if not impossible, to ascertain." Mr. Moore, however, has not even this apology for the introduction of the word *accidentally*: inasmuch as enquiries were instantly and eagerly instituted by Dr. Woodville and Mr. Wachsel, the physician and apothecary to the Small-Pox Hospital, assisted by Mr. Tanner, of the Veterinary College, until Mr. Wachsel found what they were in search of, in a place in which they expected to meet with it.

† See London Medical and Physical Journal, vol. ix. p. 309.

Mr. Moore, who is much too loose in his descriptive language, may probably mean, by a *pustular* disease, that the cow-pox is sometimes attended with *secondary vesicles*. If he doubts this, he must take the consequence of the onus under which he has placed himself of proving a negative. These secondary vesicles are certainly uncommon, but they are admitted by all the faithful reporters on vaccination, not excepting Dr. Jenner himself.

Another passage I shall feel it my duty to transcribe, as a specimen of the author's candour, as well as in vindication of myself.

"The groundless objections, (says Mr. Moore,) hitherto raised against vaccination, had, at first, a more powerful influence, than one now to be considered, which has some foundation. It was gradually observed, that a few of the multitudes who had been vaccinated were subsequently attacked with an eruptive fever. An outcry immediately ensued; some affirming, and others denying, that these eruptions were variolous. Strong attestations were signed, and virulent pamphlets were printed; for zeal and faction carried each party to extremes. But, when this fervour had a little abated, it was evident to the impartial, that both had been in the wrong.

"It was shown in the History of the Small-pox, that the same accusation was formerly raised against inoculation by Wagstaffe, De Haen, Van Swieten, and others: when the over-zealous advocates for that practice, in order to repel the charge, positively denied that the small-pox had ever attacked the same individual twice. A similar indiscretion was committed by some warm friends of vaccination.* They refused their assent to the evidence in any case where small-pox was said to have seized a person who had previously been vaccinated, and were precipitately convinced that vaccination was an infallible preventive of small-pox for life. Assertions of that force ought to have been left to the modern church of Rome: they were not in use in the ancient ritual. For Quintus Cicero,† an orthodox pagan, and a staunch believer in augury, supported this doctrine by boldly comparing it with the science of medicine; and maintained, that the predictions of a soothsayer were not more fallacious, than the prognostications of a physician. Although the aptitude of this simile is inadmissible, yet it must be granted, that infallibility is inapplicable to human nature."

---

"* Amongst whom was the author."

"† 'At nonnunquam ea, quæ prædicta sunt, minus eveniunt. Quæ tamen id ars non habet? Earum dico artium quæ conjectura continentur, et sunt opinabiles. An Medicina non putanda est? Quam tamen multa fallunt.'—*Cicer. de Divinat.* lib. i. § 14."

Those who wish to pursue this philosophical enquiry, may refer to page 67 of Mr. Moore's History: for myself, I am perfectly satisfied with his candid confession. At the same time, let me be allowed to claim an exemption from the number of those who joined in "this outcry," or who were guilty of that "indiscretion," or of that "zeal and faction," which carried each party into extremes. Four years before Dr. Jenner's first publication on the Cow-pox, I had given it as my opinion, that the susceptibility for small-pox might be so strong in some subjects as to render them susceptible of the impression from the virus a second time, of which fact, some cases appeared to me so well authenticated, that I was not sufficiently sceptical to doubt them.—(Morbid Poisons, p. 55, first edition, anno 1795.) When the cases of Fulwood's-Rents were published, I saw no reason to question the accuracy of the narration, and was not only the first, but for a long time the only one, who, without disputing the cases, still vindicated the practice of vaccination, by urging that similar events had occurred after small-pox, whether received casually or by inoculation, and reminding the public that this opinion was not taken up in consequence of any recent event, but had been by me admitted before any failures from vaccination had been suspected.*

To show that I should not only be exempted from this, but from other charges of "zeal and faction," I shall take

---

* The following passage, from a tract written in popular language in favour of vaccination, and published in 1805, for the benefit of the Small-pox Hospital, will best explain my meaning. —"This last argument [that a person may receive the small-pox twice] seems very strange to those who suppose that there are rules which admit of no exceptions. But it is well known, that some people never take the small-pox at all, and it is equally certain that some few, happily very few, have it twice. I know some say, that this was never thought of till the cow-pox made its appearance, but the following quotation is from a book published before vaccination was practised.

"'It is a law with most morbid poisons, that a constitution which has once gone through the action excited by them is no longer susceptible of it. This is the case, with very *few exceptions*, in the *small-pox*. I say with very *few exceptions*, because some cases to the contrary have been so well authenticated, that I am not sceptical enough to doubt that susceptibility may be so strong in some particular constitutions as to *admit of the disease a second time after a certain period*.'—See *Observations on Morbid Poisons*, published by Johnson, 8vo. 1795."

the liberty of transcribing a part of my letter to the College of Physicians, so long ago as the year 1806.

"The college are pleased to expect my opinion of the causes which have hitherto prevented its general adoption.

"Besides the prudent backwardness of most in admitting novelties into practice without ample proof of their utility, the causes which have prevented the general adoption of vaccination appear to me to have been principally the mistaken zeal of its friends.

"Another inconvenience has arisen from a too great forwardness at answering objections before they were sufficiently matured; hence, when variola appeared after vaccination, the event was either denied, or explained by so many minute causes as were sufficient to frighten the ignorant, disgust the candid, and induce the prudent to avoid an experiment, the result of which was not sufficiently understood.

"A practice at one time represented as so simple that the clergy and females were invited to undertake it, became at once so mysterious, that only a chosen few were said to *understand* vaccination; every untoward event was imputed to ignorance between the true and spurious pustule, to taking matter at too late a period, and to other causes still less satisfactory.

"Had these uncertainties really existed, they would have been sufficient objections against a practice, the object of which is to secure the subject from a formidable disease, and from which he might be secured by another, certainly less desirable, but well-ascertained operation. But the truth is, that vaccination is as simple as it was at first announced; that the true character of its vesicle is more certain than the local effect of any other morbid poison; that it is impossible to confound it with a pustule of any kind; and, that every difficulty might have been avoided by requiring a correct register of the progress from the period of insertion to cicatrization, or for the most part of perfect scabbing.

"I am, Sir, your obedient humble servant,

"JOSEPH ADAMS."

"*To Dr. James Hervey, &c. &c.*"

These remarks, confirmed by extracts from published works, are sufficient to show, that, though the first person who announced the cow-pox to the public, I was so far from claiming the discovery, as to deem an apology necessary for not pursuing an enquiry so important to medicine, and so immediately connected with the object of my publication on *Morbid Poisons.* 2ndly, That my apology was nothing less than a due consideration of justice to the real Discoverer. 3rdly, That I am not to be included in the number of those who considered cow-pox a pustular disease. And, lastly, that instead of joining the indiscreet zealots of either side, the object of all my writings was to repress the "indiscreet zeal" of both parties; and, by a careful examination and accurate register, to render our knowledge of the subject as perfect as a pathological fact can be rendered.

Above twenty years ago, I had occasion to remind Mr. Moore of his inattention to logical accuracy. Should he think what is now offered worth his notice, perhaps he may take the same opportunity of vindicating his doctrine of fermentation in the living body under the influence of a morbid poison.—See "*Observations on Morbid Poisons,*" &c. p. 14, edit. 1795, 8vo.

*Hatton-garden*; *March* 1818.

---

*For the London Medical and Physical Journal.*

